# Identification of IRAK1BP1 as a candidate prognostic factor in lung adenocarcinoma

**DOI:** 10.3389/fonc.2023.1132811

**Published:** 2023-03-13

**Authors:** Lei Guo, Weiping Zhou, Ziwei Xu, Xiaoqing Cao, Shiya Wan, Ying Yi Zhang, Jie Zhang, Hezhe Lu

**Affiliations:** ^1^ Life Science and Medicine, University of Science and Technology of China, Hefei, China; ^2^ State Key Laboratory of Membrane Biology, Institute of Zoology, Chinese Academy of Sciences, Beijing, China; ^3^ Institute for Stem Cell and Regeneration, Chinese Academy of Sciences, Beijing, China; ^4^ University of Chinese Academy of Sciences, Beijing, China; ^5^ Cancer Research Centre, Beijing Tuberculosis and Thoracic Tumor Research Institute, Beijing Chest Hospital, Capital Medical University, Beijing, China; ^6^ Department of Thoracic Surgery, Beijing Chest Hospital, Capital Medical University, Beijing, China; ^7^ Centre for Systems Biology, Lunenfeld-Tanenbaum Research Institute, Toronto, ON, Canada; ^8^ Department of Molecular Genetics, University of Toronto, Toronto, ON, Canada; ^9^ National Key Laboratory for Novel Software Technology, Nanjing University, Nanjing, China

**Keywords:** lung adenocarcinoma, LUAD, tumor organoid, biomarker, RNA-sequencing, DEG analysis

## Abstract

**Introduction:**

Lung cancer is one of the major causes of cancer-related mortality worldwide. High-throughput RNA sequencing (RNA-seq) of surgically removed tumors has been used to identify new biomarkers of lung cancer; however, contamination by non-tumor cells in the tumor microenvironment significantly interferes with the search for novel biomarkers. Tumor organoids, as a pre-clinical cancer model, exhibit similar molecular characteristics with tumor samples while minimizing the interference from other cells.

**Methods and Results:**

Here we analyzed six RNA-seq datasets collected from different organoid models, in which cells with oncogenic mutations were reprogrammed to mimic lung adenocarcinoma (LUAD) tumorigenesis. We uncovered 9 LUAD-specific biomarker genes by integrating transcriptomic data from multiple sources, and identified IRAK1BP1 as a novel predictor of LUAD disease outcome. Validation with RNA-seq and microarray data collected from multiple patient cohorts, as well as patient-derived xenograft (PDX) and lung cancer cell line models confirmed that IRAK1BP1 expression was significantly lower in tumor cells, and had no correlation with known markers oflung cancer prognosis. In addition, loss of IRAK1BP1 correlated with the group of LUAD patients with worse survival; and gene-set enrichment analysis using tumor and cell line data implicated that high IRAK1BP1 expression was associated with suppression of oncogenic pathways.

**Discussion:**

In conclusion, we demonstrate that IRAK1BP1 is a promising biomarker of LUAD prognosis.

## Introduction

Lung cancer is one of the most frequently diagnosed and deadliest cancers in the world (1). Comprising approximately 80% to 85% of all cases of lung cancer (2), non-small-cell lung carcinoma (NSCLC) is a heterogeneous disease that consists of tumors originating from different cell types in the lung. Some of the most frequently diagnosed subtypes of NSCLC include lung adenocarcinoma (LUAD), squamous cell carcinoma (LUSC), and large cell carcinoma, with LUAD being the most common subtype. While the gold standard of care for early-stage lung cancer is surgical resection, tumors of later stages are often treated with a combination of surgery and other treatments such as chemotherapy, targeted therapy, radiotherapy, or immunotherapy (3). Despite recent advances in the diagnosis and treatment of lung cancer, prognosis remains dire for many patients, as the early detection and prediction of long-term treatment response remain challenging. Therefore, the identification of new biomarkers that are indicative of lung cancer progression is critical for improving patient survival.

The search for biomarkers of lung cancer has benefitted largely from advances in biomedical and molecular technologies such as tumor messenger RNA (mRNA) microarray and RNA-sequencing (RNA-seq). Molecular profiling of resected lung tumors has uncovered genes indicative of lung cancer progression (4–7). However, a number of technical limitations may hinder future discoveries. For example, surgically removed tumors often contain stromal and immune cells in the tumor microenvironment (TME), which may generate ‘noise’ in sequencing and reduce the likelihood of uncovering cancer-specific biomarkers. The emergence of single cell RNA-seq (scRNA-seq) has allowed for the differentiation of tumor cells from cells in the TME (7, 8); yet partial detection of the transcriptome of different cells (a.k.a. dropouts) has made it difficult to identify novel biomarkers (9). In an attempt to address some of these challenges, tumor organoids developed from murine cells or human induced pluripotent stem-cells (iPSCs) carrying oncogenic mutations were utilized to minimize contamination from non-tumor cells while providing enough cells for performing scRNA-seq or bulk RNA-seq (10, 11). Evidently, these tumor organoids effectively recapitulated the phenotypic and epigenetic characteristics of lung tumors, demonstrating the physiological relevance of these models. Furthermore, tumor organoids serve as an excellent tool for the study of early events in tumorigenesis and cancer progression, which may have predictive power.

Here, we describe our study exploring 6 RNA-seq datasets from two studies using tumor organoids as pre-clinical models of LUAD (10, 11). We uncovered 9 genes that were differentially expressed (DEGs) across all 6 different organoid models. Interestingly, among these DEGs, we identified IL-1R-associated kinase 1 binding protein 1 (IRAK1BP1), an anti-inflammatory factor that negatively regulates Toll-like receptor (TLR) signaling (12–14). While studies have elucidated the anti-inflammatory function of IRAK1BP1, the association between IRAK1BP1 and cancer has not been reported and its role in cancer is unknown. Further investigation of IRAK1BP1 expression in publicly available LUAD patient data found that IRAK1BP1 expression was lower in tumor samples relative to the corresponding normal tissue; and its predictive power is independent from other known indicators of patient outcome, such as known oncogenic mutations, tumor mutation burden, age or gender. Importantly, loss of IRAK1BP1 coincided with the group of LUAD patients with poorer prognosis; and gene-set enrichment analysis (GSEA) in both cell line and patient data suggested that elevated IRAK1BP1 expression was associated with reduced expression of oncogenic pathways. Our analysis suggests that IRAK1BP1 is an independent prognostic factor of LUAD that robustly predicts LUAD tumor progression, which may be valuable for future development of personalized treatments.

## Materials and methods

### RNA-seq analysis of mouse organoids

Gene expression of Mouse Organoids was retrieved from the NCBI GEO database (https://www.ncbi.nlm.nih.gov/geo/). Six RNA-seq datasets from two studies, GSE150425 (15) and GSE180360 (11), were downloaded from the GEO website. Differential expression analysis was performed using the “edgeR” R package (version 3.40.0). Genes with adjusted P value < 0.05 and |log2FC| > 0.5 were identified as differentially expressed genes (DEGs).

### Gene expression and prognosis analysis

**Figure 1:**
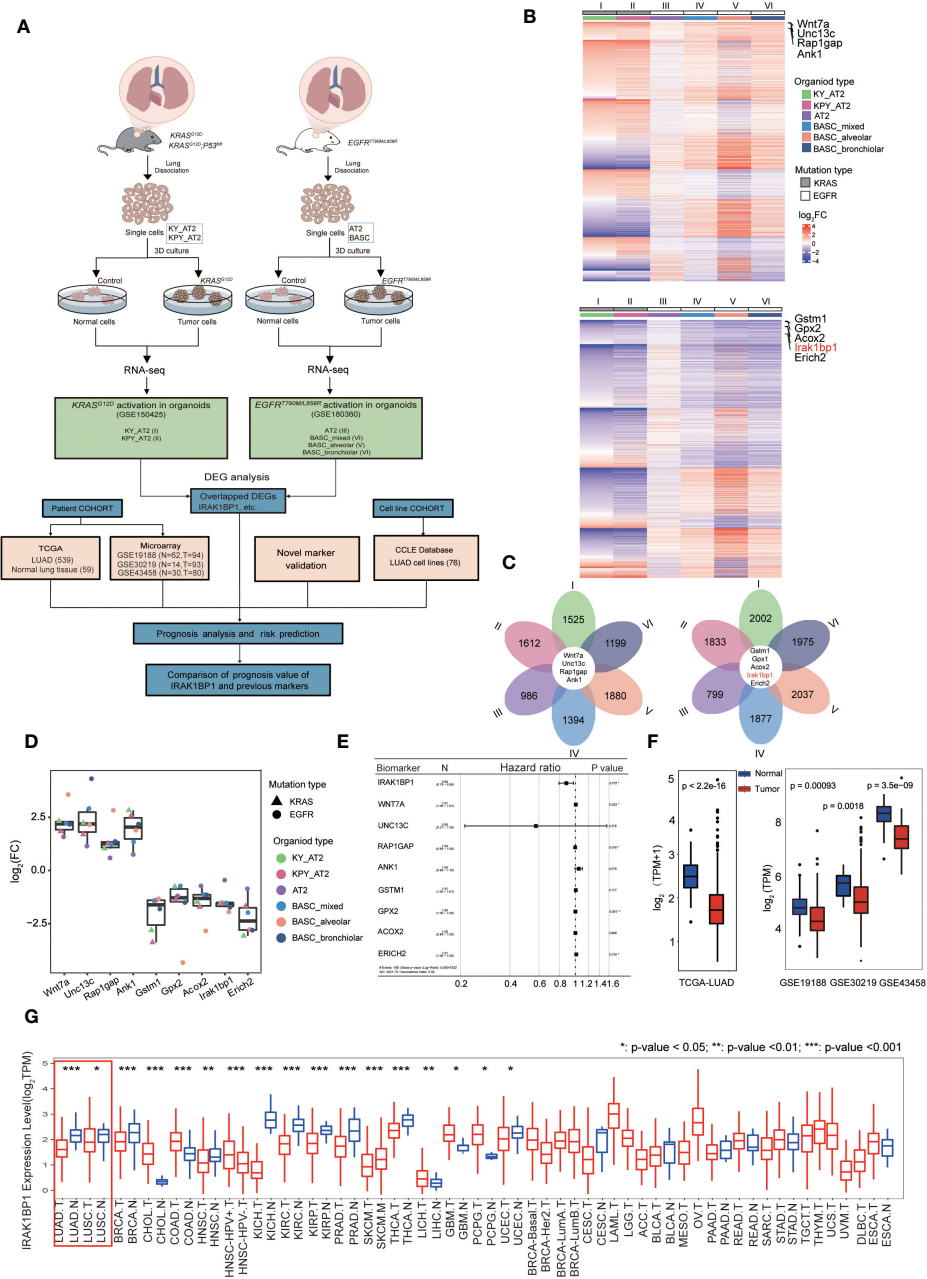
IRAK1BP1 is downregulated in six different organoid types. **(A)** Schematic describing the workflow for identifying NSCLC-specific DEGs from publicly available RNA-sequencing data from organoids with the indicated oncogenic mutations and cell of origin. FACS sorting, *in vitro* propagation, activation of KRAS or EGFR were performed for lung cells harvested from mice with the indicated genotype. In the KRAS-driven lung cancer, organoids were developed from dissected lungs of adult *KRAS^LSL-G12D^*; *ROSA26^LSL-YFP^* (KY), *KRAS^LSL-G12D^*; *P53^fl/fl^*;*ROSA26^LSL-YFP^* (KPY) mice and used FACS to isolate alveolar type 2 (AT2) cells. In the EGFR-driven lung cancer, organoids were developed from the dissecting lungs and tracheas of *EGFR^T790M/L858R^* mice and used FACS to isolate AT2 cells and bronchioalveolar stem cells (BASCs). Organoids derived from BASCs were manually subcloned into alveolar, bronchiolar, and mixed cultures by their microscopic morphologies. Both of alveolar and bronchiolar organoids are contained in mixed BASCs. **(B)** Heatmap of DEGs that were expressed in six models. Top: the total upregulated genes; bottom: the total downregulated genes. **(C)** Petal diagrams showing the numbers of differentially expressed genes in each dataset and their overlap. Left: the numbers of up-regulated genes in the six models; right: the numbers of down-regulated genes in the six models. **(D)** Symbols showing the fold-change in log2FC of common DEGs in all 6 datasets and means ± SD are indicated. **(E)** Multivariable Cox regression of candidate LUAD biomarker genes in the TCGA LUAD cohort (n = 539). **(F)** The expression of IRAK1BP1 in LUAD and corresponding normal lung tissues. **(G)** The differential expression of IRAK1BP1 between normal tissues and tumor tissues in various cancers. *P < 0.05, **P < 0.01, and ***P < 0.001.

TIMER (http://timer.cistrome.org/) was used to analyze the differential expression of IRAK1BP1 between normal tissues and tumor types (**Figure 1G**). [IRAK1BP1 expression levels in various tumor types and the corresponding normal tissues were analyzed by TIMER (http://timer.cistrome.org/) (**Figure 1G**)]. The clinical characteristics, somatic mutations and RNA-seq data in TPM (transcripts per million reads) of lung cancer patients, as well as the corresponding normal lung tissue data in the cancer genome atlas (TCGA) (https://portal.gdc.cancer.gov/) were downloaded using TCGAbiolinks (16). The correlation between IRAK1BP1 expression and overall survival (OS) was analyzed by the “survival” R package (version 3.4-0). Patient samples were divided into four groups based on 25%, 50% and 75% quartiles ranked by IRAK1BP1 expression, and the Kaplan–Meier survival analysis for OS was performed in the top and bottom groups. Statistical significance of survival curves was assessed by a log-rank test.

### Correlation analysis of gene mutations and LUAD clinical data

We calculated the percentage of mutation for each gene across all the LUAD samples and selected the top 20 genes with the highest mutation rates. We then ranked the samples based on the expression of IRAK1BP1 from high to low, and split all cases into three groups (high, < 25%; medium, ≥ 25% and ≤ 75%; low, > 75%). A waterfall plot was displayed using the “maftools” R package (version 2.14.0).

### Function and gene set enrichment analysis

Gene expression data of LUAD cell lines were retrieved from the cancer cell line encyclopedia (CCLE) (https://sites.broadinstitute.org/ccle/). Differentially expressed genes in LUAD samples from TCGA and LUAD cell lines from CCLE were identified with adjusted P value < 0.05 and |log2FC| > 1. Gene Ontology enrichment analyses were performed by the “clusterProfiler” R package (version 4.5.2). The “fgsea” R package (version 1.22.0) was used to perform gene set enrichment analysis (GSEA).

## Results

### Analysis of RNA-seq data obtained from organoid models identified LUAD-specific DEGs and IRAK1BP1, which is down-regulated in six organoid models as well as in LUAD patient samples

To search for novel biomarkers for lung cancer that predict disease outcome, we analyzed RNA-seq datasets from two studies using murine tumor organoids derived from murine alveolar epithelial progenitor (AT2) cells and/or bronchioalveolar stem cells (BASCs), which were postulated to give rise to LUAD (10). **Figure 1A** demonstrates the workflow of RNA-seq data collection from mouse organoids with or without the *in vitro* induced activation of *KRAS^G12D^* (10) or *EGFR^T790M^* ^/^ *^L858R^* (11), which are two of the most predominant oncogenic mutations in NSCLC (17); this was then followed by DEG analysis to find genes aberrantly expressed in tumor organoids by comparing the transcriptomic profiles of tumor versus normal (control) organoids. Data collected for a total of six organoid types were analyzed for our study (**Figure 1A**). Lists of DEGs for each dataset were then compared to find 9 DEGs that were common to all six organoid models of LUAD (**Figures 1A-C**). Genes that were consistently up-regulated across all LUAD organoid types include *Wnt7a*, *Unc13c*, *Rap1gap*, and *Ank1* (**Figure 1B**, upper heatmap), while genes consistently down-regulated were *Gstm1*, *Gpx2*, *Acox2*, *Irak1bp1*, and *Erich2* (**Figure 1B**, bottom heatmap). The log fold change (log2FC) for each DEG was displayed in **Figure 1D**, showing that these 9 genes were indeed consistently up- or down-regulated in all 6 organoid types that were analyzed.

To find candidate biomarkers that may be valuable to the prediction of LUAD progression, we retrieved and analyzed LUAD patient transcriptomic and prognostic data from the cancer genome atlas (TCGA) as well as 3 published tumor microarray datasets (GSE19188, GSE30219, GSE43458) to obtain our list of shared DEGs. To see how well these candidate biomarkers predict patient outcome, we measured the hazard ratio (hr) of the 9 DEGs discovered, and found that one gene, in particular, called IRAK1BP1 had a lower hr value with statistical significance, suggesting that it had greater predictive power than the rest of the DEGs (**Figure 1E**). As a result, we decided to further investigate the expression of IRAK1BP1 in patient samples from TCGA as well as 3 independent cohorts; our findings suggested that IRAK1BP1 expression in LUAD patients resembled what was observed in the organoid models, showing reduced expression in tumor tissue relative to normal tissue (**Figure 1F**).

Interestingly, the differential expression of IRAK1BP1 is also observed in other cancer types: we assessed IRAK1BP1 expression for all cancer types with available data (**Figure 1G**). The differential expression of IRAK1BP1 is again evident in LUAD and LUSC, the two main NSCLC subtypes (red box); IRAK1BP1 expression is lower in tumor relative to normal tissue in many other cancer types such as breast cancer (BRCA), kidney cancers (KICH, KIRC), and prostate adenocarcinoma (PRAD); while in some cancer types, such as cholaniocarcinoma (CHOL) and colon adenocarcinoma (COAD), the opposite is true (**Figure 1G**). The differential expression of IRAK1BP1 in various cancer types warrants further survival analysis in cancers other than lung cancer.

### IRAK1BP1 expression correlates with LUAD patient survival and is an independent marker of LUAD prognosis

**Figure 2:**
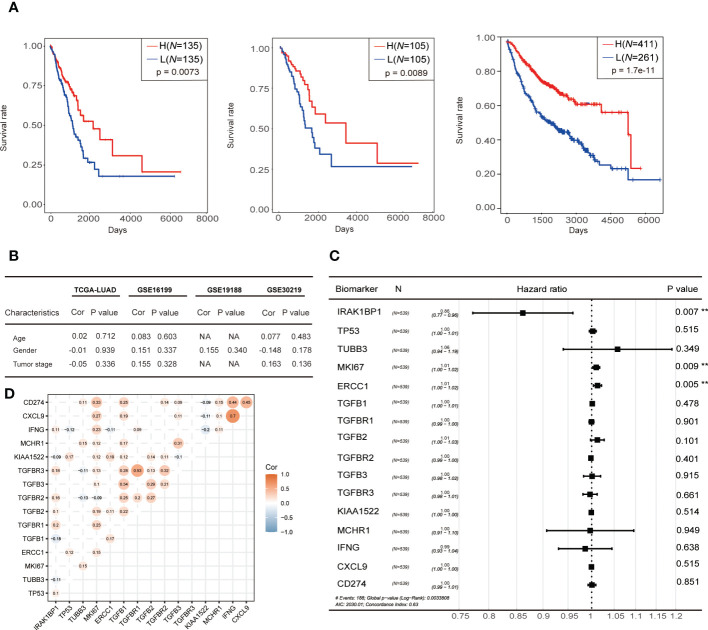
Correlation between IRAK1BP1 expression and prognosis. **(A)** The overall survival curve of IRAK1BP1 in total LUAD patients in the high-/low-expression group. Statistical significance of survival curves was assessed by a log-rank test. The overall survival curve of IRAK1BP1 in early stage (stage I and stage II) LUAD patients in the high-/low-expression group. Statistical significance of survival curves was assessed by a log-rank test. The overall survival curve of IRAK1BP1 in LUAD patients (CAARRY, GSE14814, GSE19188, GSE29013, GSE30219, GSE31210, GSE3141, GSE31908, GSE37745, GSE43580, GSE4573, GSE50081, GSE8894) in the high-/low-expression group. Statistical significance of survival curves was assessed by a log-rank test. **(B)** Correlation between the expression of IRAK1BP1 and patient age, gender, and tumor stage in TCGA-LUAD and 3 independent patient cohorts. **(C)** The Hazard ratio of reported novel prognostic biomarkers associated with lung cancer. Statistical significance of Hazard ratio was assessed by a log-rank test. **(D)** Correlation between the expression of IRAK1BP1 and the expression of genes in **(C)**. The P < 0.05 was discarded between two genes.

We further investigated whether differential IRAK1BP1 expression correlated with differential LUAD prognosis by plotting the survival curves for LUAD patients divided into two groups (top and bottom quartiles), based on the level of IRAK1BP1 expression (**Figure 2A**). The overall survival (OS) and early-stage survival for LUAD patients in the TCGA cohorts (TCGA-LUAD), as well as the survival curves calculated from patient microarray data, suggested that LUAD patients with high IRAK1BP1 expression (red) had higher survival rates hence better prognosis compared with patients with low IRAK1BP1 (blue) (**Figure 2A**, respectively). To see how well IRAK1BP1 alone performs as a prognostic predictor relative to other known biomarkers of NSCLC prognosis, we measured the coefficients of correlation between IRAK1BP1 expression and well-studied indicators of lung cancer prognosis such as patient age, gender, and tumor stage at diagnosis, and found that IRAK1BP1 expression had no correlation with any of these factors; our analysis showed that IRAK1BP1 alone was able to robustly predict LUAD patient prognosis (**Figure 2B**). In addition, pair-wise correlation between each of a collective set of lung cancer prognostic marker genes and IRAK1BP1 expression was measured and shown in **Figure 2D**, where the color represents the coefficient of correlation and the size of the dot represents statistical significance; again, our analysis suggests that IRAK1BP1 had very weak correlation with most known biomarkers and was able to act alone as a prognostic predictor (**Figure 2D**).

To compare the performance of IRAK1BP1 as a prognostic predictor with known biomarkers of LUAD, hazard ratios (hr) were calculated for IRAK1BP1 along with previously described lung cancer biomarkers listed in **Figure 2C**: hr values lower than 1 indicates low hazards (i.e. better prognosis); the lower the hr value, the better the prognosis it predicts. Our analysis showed that IRAK1BP1 was able to robustly predict LUAD patient prognosis, outperforming each of the known biomarkers in the list (**Figure 2C**).

**Figure 3:**
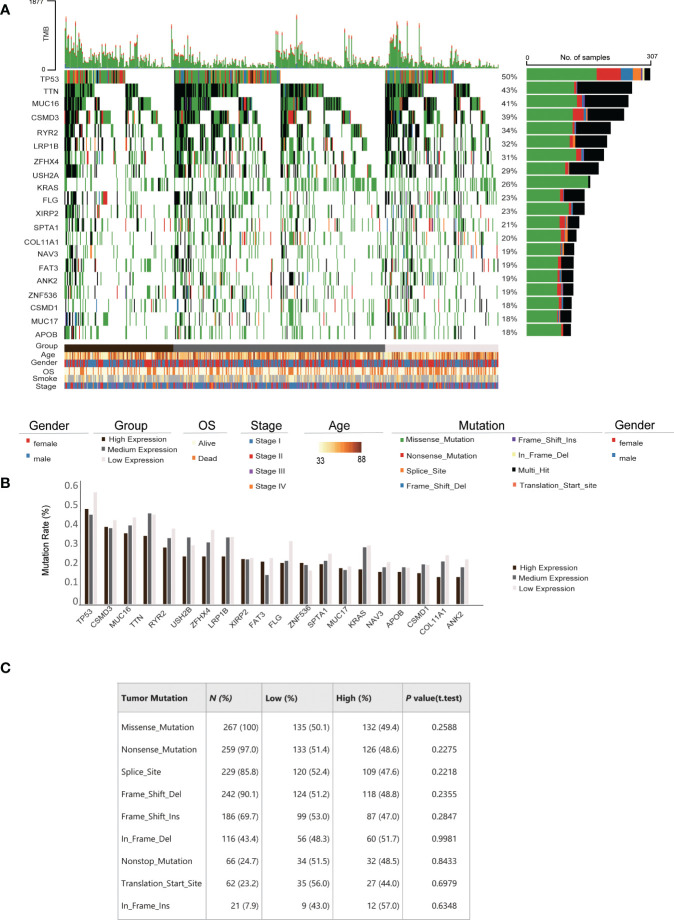
Correlation between IRAK1BP1 expression and mutation frequency in LUAD. **(A)** Distribution and type of top 20 genes with the highest mutation rates across all LUAD samples. Percentages of samples with mutations for each gene are shown at the left. Samples ranked by the expression of IRAK1BP1 from high to low (high, < 25% quantile; medium, ≥ 25% quantile and ≤ 75% quantile; low, > 75% quantile). The clinical characteristics, including age, gender, overall survival (OS), smoking history and stage, are shown at the bottom. **(B)** Mutation rates of genes in **(A)**. For each gene, the mutation rate was calculated in high, medium and low expression of IRAK1BP1, respectively. **(C)** Relationship between IRAK1BP1 expression levels in **(A)** and tumor mutation features in lung adenocarcinoma in the TCGA database.

Tumor mutation burden (TMB) and mutation rates are well-known biomarkers of lung cancer patient treatment response and prognosis. To assess whether the correlation between IRAK1BP1 expression and patient survival is a function of TMB and mutation rates, we evaluated the correlation between IRAK1BP1 expression and a number of factors including TMB, the mutation status of the top 20 genes with the highest mutation rates, age, gender, OS, smoking status, and pathological stage of LUAD (**Figure 3**). Patient samples were divided into three groups based on the level of IRAK1BP1 expression and were aligned horizontally from high to low expression (**Figure 3A**). Here, we did not observe any significant correlation between IRAK1BP1 expression and most of the factors evaluated; mutation rates were not dramatically altered for the 20 most mutated genes selected, though mutation rates in the high-expression group were slightly lower than those in low- and/or medium-expression groups (**Figure 3B**). The frequencies of different types of mutations found in LUAD patients were also fairly even between the High-IRAK1BP1 and Low-IRAK1BP1 groups (**Figure 3C**). Our results suggest that IRAK1BP1 expression correlates with LUAD prognosis and is able to act independently as a candidate predictor of disease outcome.

### GSEA and GO term analyses suggest that higher IRAK1BP1 expression is associated with suppressed oncogenic pathway signatures

**Figure 4:**
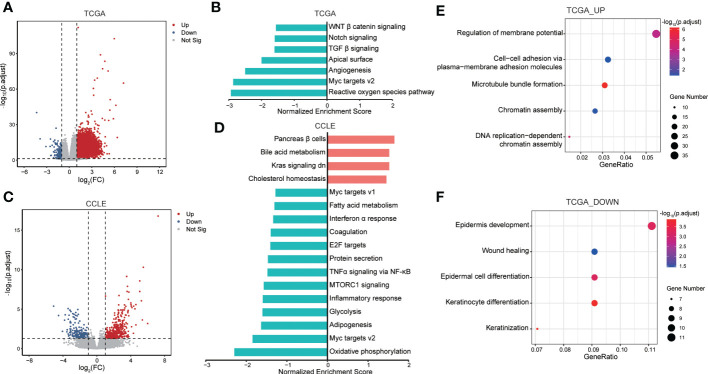
Functional enrichment analysis for prognosis signature. **(A)** Volcano plot showing DEGs between groups with high IRAK1BP1 expression and low IRAK1BP1 expression in TCGA-LUAD primary tumor samples. Red indicates elevated expression while blue indicates reduced expression (down). **(B)** GSEA enrichment analysis identified down-regulated oncogenic pathways in samples with high IRAK1BP1 expression relative to the group with low IRAK1BP1 expression in TCGA-LUAD cohort. **(C)** Volcano plot showing DEGs between groups with high IRAK1BP1 expression and low IRAK1BP1 expression in LUAD cell line of CCLE database. Red indicates elevated expression while blue indicates reduced expression (down). **(D)** GSEA enrichment analysis identified down-regulated oncogenic pathways in samples with high IRAK1BP1 expression relative to the group with low IRAK1BP1 expression in lung cancer cell lines in CCLE database. **(E)** GO analysis of up-regulated genes in the group with high IRAK1BP1 expression in TCGA-LUAD. **(F)** GO analysis of down-regulated genes in the group with high IRAK1BP1 expression in TCGA-LUAD.

To find out how IRAK1BP1 may assist in tumor formation and progression, we performed gene-set enrichment analysis (GSEA) as well as gene ontology analysis for LUAD samples in the high-IRAK1BP1 group (**Figure 4**). By comparing transcriptomes between LUAD patients with high- and low-IRAK1BP1 expression, we found genes that were up-regulated or down-regulated in patients with high IRAK1BP1 (**Figure 4A**). GSEA analysis in the high-IRAK1BP1 transcriptomes from the TCGA-LUAD patient cohorts suggested that some of the most well-known oncogenic pathways, such as the Notch, canonical Wnt, as well as the TGF-β pathways, were down-regulated in LUAD tumors from the high-IRAK1BP1 group (**Figure 4B**). Further validating the predictive power of IRAK1BP1, our GSEA analysis of high-IRAK1BP1 expressers using RNA-seq data from lung cancer cell lines in the cancer cell line encyclopedia (CCLE) database showed remarkable similarity to the GSEA results in patient data (**Figures 4C, D**). Interestingly, in addition to down-regulated oncogenic pathways, signatures of inactive KRAS pathway were also present in the high-IRAK1BP1 group (**Figure 4D**). GO term analysis of up-regulated genes (**Figure 4E**) and down-regulated genes (**Figure 4F**) in TCGA-LUAD samples with high IRAK1BP1 suggested that high IRAK1BP1 expression was associated with suppression of pathways associated with immune/inflammatory response as well as developmental/wound healing, possibly reflecting reduced TMB in this group (**Figure 4F**); in contrast, pathways associated with cytoskeleton and cell adhesion were up-regulated in the high-IRAK1BP1 group, suggesting that these cells may have better structural stability (**Figure 4E**). These findings prompt further studies to better characterize the association of IRAK1BP1 with tumorigenesis and cancer progression.

## Discussion

Recent advances in translational research and the invention of new therapeutic approaches have shed light on the diagnosis and treatment of cancer. However, many patients continue to suffer from the lack of treatment response, the development of drug resistance, and the dread of recurrence after remission. For most cancer patients, diagnosis at an earlier stage of tumor formation and/or progression means better chance of surviving. As a result, there has been ongoing effort dedicated to the search for biomarkers that enable more sensitive detection of early-stage cancers as well as more accurate prediction of disease outcome. Serving as a pre-clinical model of cancer, tumor organoids allow us to overcome the limitations of animal and patient-derived xenograft models by capturing early events of tumorigenesis and/or the initiating steps in cancer progression. While some organoid models arguably lack components from the TME and therefore may not reveal the intricacies of cancer-stroma/immune interactions, they nonetheless provide a relatively controlled system for the discovery of biomarkers and/or druggable targets.

Here, using RNA-seq data that are publicly available, we identified IRAK1BP1 as a putative prognosis predictor of LUAD disease outcome. Our analyses showed that IRAK1BP1 is not only one of the 9 LUAD-specific DEGs that are common to the 6 datasets that were analyzed, but also possesses predictive potential, as the reduced expression of IRAK1BP1 alone in tumor samples predicts poor survival. Our findings suggest that elevated IRAK1BP1 is associated with suppressed oncogenic signatures in both lung cancer cell lines and patient data, further showing promise for IRAK1BP1 as a predictor of lung cancer prognosis.

The association of IRAK1BP1 with cancer is a novel discovery, while the function of IRAK1BP1 in cancer remains to be explored. To-date, multiple studies have suggested that IRAK1BP1 promotes nuclear translocation of NF-kappaB thereby inhibiting inflammatory responses (12–14). In the context of cancer, chronic inflammation is often associated with tumor development (18); therefore it would be interesting to test if the reduced expression of IRAK1BP1 in tumor cells leads to elevated inflammatory signals and contributes to cancer progression. Mechanistic studies will allow us to evaluate the therapeutic potential of this gene.

## Data availability statement

The datasets presented in this study can be found in online repositories. The names of the repository/repositories and accession number(s) can be found in the article/supplementary material.

## Ethics statement

Ethical review and approval was not required for the study on human participants in accordance with the local legislation and institutional requirements. Written informed consent for participation was not required for this study in accordance with the national legislation and the institutional requirements. Ethical review and approval were not required for the animal study because the study was based on bioinformatic analysis of published patient data, for which ethical reviews were conducted to obtain approval. All the datasets and their source projects were properly referenced.

## Author contributions

HL, JZ and YZ conceived the project, LG and WZ performed bioinformatics and statistical analyses. ZX, XC and SW performed database collection. YZ and HL wrote the manuscript. All authors contributed to the article and approved the submitted version.

## References

[1] Siegel RL Miller KD Fuchs HE Jemal A. Cancer statistics. CA Cancer J Clin (2022) 72(2022):7–33. doi: 10.3322/caac.21708 35020204

[2] Araujo LH Horn L Merritt RE Shilo K Xu-Welliver M Carbone DP. Ch. 69 - Cancer ofthe Lung: Non-small cell lung cancer and small cell lung cancer. In: Niederhuber JE Armitage JO Doroshow JH Kastan MB Tepper JE editors. Abeloff’s Clinical Oncology, 6th ed. Philadelphia, PA: Elsevier (2020).

[3] Petrella F Rizzo S Casiraghi M Bardoni C Mohamed S Musso V. State of the art and new perspectives in surgical treatment of lung cancer: A narrative review. Transl Cancer Res (2022) 11:3869–75. doi: 10.21037/tcr-22-1491 PMC9641123 36388035

[4] Ma Q Xu Y Liao H Cai Y Xu L Xiao D. Identification and validation of key genes associated with non-small-cell lung cancer. J Cell Physiol (2019) 234:22742–52. doi: 10.1002/jcp.28839 31127628

[5] Lai Y-H Chen W-N Hsu T-C Lin C Tsao Y Wu S. Overall survival prediction of non-small cell lung cancer by integrating microarray and clinical data with deep learning. Sci Rep (2020) 10:4679. doi: 10.1038/s41598-020-61588-w 32170141 PMC7069964

[6] Huang H Yu H Li X Li Y Zhu G Su L. Genomic analysis of TNF-related genes with prognosis and characterization of the tumor immune microenvironment in lung adenocarcinoma. Front Immunol (2022) 13. doi: 10.3389/fimmu.2022.993890 PMC9732939 36505472

[7] Li Q Wang R Yang Z Li W Yang J Wang Z. Molecular profiling of human non-small cell lung cancer by single-cell RNA-seq. Genome Med (2022) 14:87. doi: 10.1186/s13073-022-01089-9 35962452 PMC9375433

[8] Wu F Fan J He Y Xiong A Yu J Li Y. Single-cell profiling of tumor heterogeneity and the microenvironment in advanced non-small cell lung cancer. Nat Commun (2021) 12:2540. doi: 10.1038/s41467-021-22801-0 33953163 PMC8100173

[9] Qiu P. Embracing the dropouts in single-cell RNA-seq analysis. Nat Commun (2020) 11:1169. doi: 10.1038/s41467-020-14976-9 32127540 PMC7054558

[10] Dost AFM Moye AL Vedaie M Tran LM Fung E Heinze D. Organoids model transcriptional hallmarks of oncogenic KRAS activation in lung epithelial progenitor cells. Cell Stem Cell (2020) 27:663–678.e8. doi: 10.1016/j.stem.2020.07.022 32891189 PMC7541765

[11] Chen F Liu J Flight RM Naughton KJ Lukyanchuk A Edgin AR. Cellular origins of EGFR-driven lung cancer cells determine sensitivity to therapy. Adv Sci (Weinh) (2021) 8:e2101999. doi: 10.1002/advs.202101999 34622577 PMC8596110

[12] Smirnova II Conner JR Poltorak A. Forward genetic analysis of toll-like receptor responses in wild-derived mice reveals a novel antiinflammatory role for IRAK1BP1. J Exp Med (2008) 205:305–14. doi: 10.1084/jem.20071499 PMC2271017 18268037

[13] Smirnova II Conner JR Moseman AP Poltorak A. IRAK1BP1 inhibits inflammation by promoting nuclear translocation of NF-kappaB p50. Proc Natl Acad Sci USA (2010) 107:11477–82. doi: 10.1073/pnas.1006894107 PMC2895073 20534545

[14] Li H Lessard CJ Adrianto I Ice JA Rasmussen A Grundahl KM. Variants at multiple loci implicated in both innate and adaptive immune responses are associated with sjögren's syndrome. Nat Genet (2013) 45:1284–92. doi: 10.1038/ng.2792 PMC3867192 24097067

[15] Shi R Radulovich N Ng C Liu N Notsuda H Cabanero M. Organoid cultures as preclinical models of non-small cell lung cancer. Clin Cancer Res (2020) 26:1162–74. doi: 10.1158/1078-0432.CCR-19-1376 31694835

[16] Colaprico A Silva TC Olsen C Garofano L Cava C Garolini D. TCGAbiolinks: An R/Bioconductor package for integrative analysis of TCGA data. Nucleic Acids Res (2016) 44:e71. doi: 10.1093/nar/gkv1507 26704973 PMC4856967

[17] Collisson EA Campbell JD Brooks AN Berger AH Lee W Chmielecki J. Comprehensive molecular profiling of lung adenocarcinoma. Nature (2014) 511:543–50. doi: 10.1038/nature13385 PMC4231481 25079552

[18] Wu L Zhao H Yan G Chen Y Zhou M Wu Y. Inflammation and tumor progression: Signaling pathways and targeted intervention. Signal Transduct Target Ther (2021) 6:263. doi: 10.1038/s41392-021-00658-5 34248142 PMC8273155

